# Deletion of Intragenic Tandem Repeats in Unit C of *FLO1* of *Saccharomyces cerevisiae* Increases the Conformational Stability of Flocculin under Acidic and Alkaline Conditions

**DOI:** 10.1371/journal.pone.0053428

**Published:** 2013-01-04

**Authors:** Ee Li, Feng Yue, Qi Chang, Xuena Guo, Xiuping He, Borun Zhang

**Affiliations:** 1 Institute of Microbiology, Chinese Academy of Sciences, Beijing, China; 2 University of Chinese Academy of Sciences, Beijing, China; Université de Nice-CNRS, France

## Abstract

Flocculation is an attractive property for *Saccaromyces cerevisiae*, which plays important roles in fermentation industry and environmental remediation. The process of flocculation is mediated by a family of cell surface flocculins. As one member of flocculins, Flo1 is characterized by four families of repeats (designated as repeat units A, B, C and D) in the central domain. It is generally accepted that variation of repeat unit A in length in Flo1 influences the degree of flocculation or specificity for sugar recognization. However, no reports were observed for other repeat units. Here, we compared the flocculation ability and its sensitivity to environmental factors between yeast strain YSF1 carrying the intact *FLO1* gene and yeast strains carrying the derived forms of *FLO1* with partial or complete deletion of repeats in unit C. No obvious differences in flocculation ability and specificity of carbohydrate recognition were observed among these yeast strains, which indicates the truncated flocculins can stride across the cell wall and cluster the N-terminal domain on the surface of yeast cells as the intact Flo1 thereby improving intercellular binding. However, yeast strains with the truncated flocculins required more mannose to inhibit completely the flocculation, displayed broad tolerance of flocculation to pH fluctuation, and the fewer the repeats in unit C, the stronger adaptability of flocculation to pH change, which was not relevant to the position of deletion. This suggests that more stable active conformation is obtained for flocculin by deletion the repeat unit C in the central domain of Flo1, which was validated further by the higher hydrophobicity on the surface of cells of YSF1c with complete deletion of unit C under neutral and alkaline conditions and the stabilization of GFP conformation by fusion with flocculin with complete deletion of unit C in the central domain.

## Introduction

Yeast flocculation is described as a reversible, asexual and calcium dependent process, in which cells adhere to form flocs consisting of thousands of cells and separate from the bulk medium by sedimentation or by rising to the surface [1]. Flocculation is an attractive property for yeast since it provides an effective, environment-friendly, simple and cost-free way to separate yeast cells from the culture broth at the end of fermentation [2]. Moreover, flocculation is also a cooperative protection mechanism that shields cells from stressful environments [3]. Recently, a flocculent yeast strain was used to remove heavy metals (Cu^2+^, Ni^2+^ and Zn^2+^) from a synthetic effluent based on its flocculation ability [4], which makes flocculent yeast employed as biological “scrubbers” possible [1].

Flocculation in *Saccaromyces cerevisiae* is mediated by specific cell surface proteins, flocculins, which are capable of binding directly to mannose residues present on the cell wall of adjacent yeast cells [5]. According to different sugar inhibition, two main flocculation phenotypes have been classified: Flo1-type which is mannose-sensitive only and NewFlo-type which is sensitive to glucose, maltose, sucrose as well as mannose [6], [7]. These two types of flocculation are all under genetic control and influenced by environmental factors, such as pH and ionic strength [8], [9]. Different chromosomal genes (*FLO1*, *FLO5*, *FLO8*, *FLO9*, *FLO10*, *FLO11*, *FLONS*, *FLONL* and *Lg-FLO1*) related to flocculation of *S. cerevisiae* have been identified and all described as dominant genes [10], [11], [12], [13], [14], [15]. Proteins encoded by these *FLO* genes share a common modular organization that consists of three domains: an amino-terminal lectin domain which protrudes from the cell surface and is responsible for the binding to carbohydrate, a central domain that is extremely rich in serine and threonine residues, and a carboxyl-terminal domain containing a glycosyl phosphatidylinositol anchoring sequence [1]. DNA sequence responding to the central domain contains many tandem repeat regions, which are highly dynamic components of yeast genome. These repeats drive slippage and recombination reactions within and between *FLO* genes, leading to the generation of novel *FLO* alleles or pseudogenes, which endows yeast cells with diversity and variety in flocculation ability [16], [17]. *FLO1*, the best-known flocculation gene in yeast, contains an open reading frame of 4614 bp encoding for a protein of 1537 amino acids, which shares the common three-domain structure with other flocculation proteins. The large central domain of flocculin Flo1 is characterized by four families of serine- and threonine-rich repeats: eighteen tandem repeats of 45 amino acid residues (repeat unit A), two repeats of 20 amino acid residues (repeat unit B), three repeats of 51 amino acid residues (repeat unit C) and three repeats of 9 amino acid residues (repeat unit D). Due to the tandem repeat sequences, *FLO1* is unstable in genetics and evolves in nature rapidly [17]. It has been discovered that flocculation of yeast cells tended to decrease with successive generations, while other properties were generally unchanged [18]. Various truncated forms of *FLO1*, such as *FLO1S, FLO1M, FLO1G, FLONS* and *FLONL*, were isolated from yeast by the method of genome library [14], [19], [20], [21]. Sequence analysis indicated that the deletion in these truncated forms occurred only in the tandem repeat unit A of *FLO1*. Meanwhile, the number variation of repeats in unit A of *FLO1* influences the degree of flocculation, the more the repeats, the stronger the flocculation [17].

Apart from repeat unit A, other three repeat units (B, C and D) also locate in the central domain of flocculin, which may also influence the conformation and function of Flo1. The phenomenon that flocculation of yeast strains carrying the derived forms of *FLO1* with complete deletion of repeat unit B or unit D was more tolerant to pH or mannose variation in environment than that of yeast strain with the intact *FLO1* was observed in our previous study [22]. In the present study, the influence of deletion of repeat unit C in *FLO1* gene on the function and stability of flocculin was investigated.

## Results

### Flocculation of Yeast Strains Carrying the Intact *FLO1* or its Derived Forms

**Figure 1:**
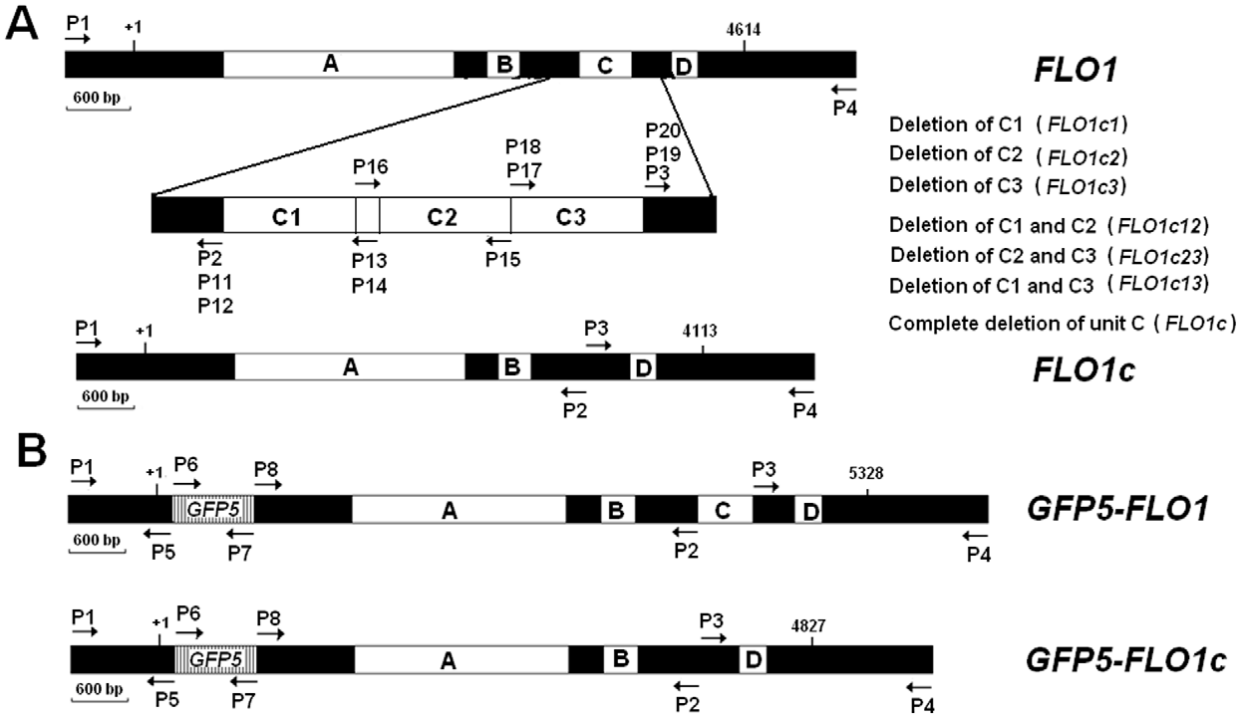
Schematic diagram of intact *FLO1* and its derived forms (A), and the fusion expression cassettes of *GFP5* with *FLO1* or *FLO1c* (B). Sequence from +1 to 4614 bp in *FLO1* is the intact ORF. Regions A, B, C and D are the tandem repeat units according to the features of Flo1 deposited in SIB Bioinformatics Resource Portal (http://expasy.org). The sites for primers used in this study were indicated.

The 6082 bp of intact flocculation gene *FLO1* was cloned from *S. cerevisiae* YS59 by PCR with a primer set of P1 and P4. The derived forms of *FLO1* with partial or complete deletion of repeat unit C were constructed via fusion PCR using primers listed in Table 1 and Figure 1A, and confirmed by sequence analysis and alignment. The nonflocculent *S. cerevisiae* YS58 was transformed with the empty vector YCp50 and recombinant plasmids pYCF1, pYCF1c, pYCF1c1, pYCF1c2, pYCF1c3, pYCF1c12, pYCF1c13, and pYCF1c23 respectively to generate recombinant yeast strains YSP50, YSF1, YSF1c, YSF1c1, YSF1c2, YSF1c3, YSF1c12, YSF1c13 and YSF1c23. The recombinant strains were checked by plasmid recovery and PCR analysis. After 10 generations culture of recombinant strains in YPD medium, all of the tested single colonies for each strain were able to grow on SD medium containing leucine, histidine and tryptophan, which indicated that plasmids in yeast cells were stable genetically.

**Table 1:** Primers used in this study.

Primers	Sequences (5′ to 3′)*	Purposes
P1	Tttgtcgacggcttccagtatgctttcac	P1/P4: *FLO1*
P2	Gactcttcattcgcggtagcaggtggtaatg	P1/P2+ P3/P4: complete deletion of unit C in *FLO1*
P3	ttaccacctgctaccgcgaatgaagagtctgtcag	
P4	Acaagctttactacacttcctgggaacg	
P5	agttcttctcctttactcatggctcctgaggccacacactag	For GFP tagged flocculins
P6	ctagtgtggcctcaggagccatgagtaaaggagaagaact	For GFP tagged flocculins
P7	taagcacgcctctgttttgtatagttcatccatgc	For GFP tagged flocculins
P8	atgaactatacaaaacagaggcgtgcttaccagc	For GFP tagged flocculins
P11	tgctttgttgtctcggtagcaggtggtaatg	P1/P11+ P16/P4: deletion of C1 in unit C of *FLO1*
P12	gcctcgattctgtggtggtagcaggtggtaatg	P1/P12+ P18/P4: deletion of C1 and C2 in unit C of *FLO1*
P13	gcctcgattctgtggtttgctctgttgtccctttgg	P1/P13+ P17/P4: deletion of C2 in unit C of *FLO1*
P14	gactcttcattcgcttgctctgttgtccctttgg	P1/P14+ P20/P4: deletion of C2 and C3 in unit C of *FLO1*
P15	gactcttcattcgcggaaataggacaccatgttg	P1/P15+ P19/P4: deletion of C3 in unit C of *FLO1*
P16	ttaccacctgctaccgagacaacaaagcaaacc	P1/P11+P16/P15+P19/P4: deletion of C1 and C3 in unit C of *FLO1*
P17	agggacaacagagcaaaccacagaatcgaggcaac	
P18	ttaccacctgctaccaccacagaatcgaggcaac	
P19	atggtgtcctatttccgcgaatgaagagtctgtcag	
P20	agggacaacagagcaagcgaatgaagagtctgtcag	

* Restriction sites of *Sal*I and *Hin*dIII in primers P1 and P4 are underlined.

**Figure 2:**
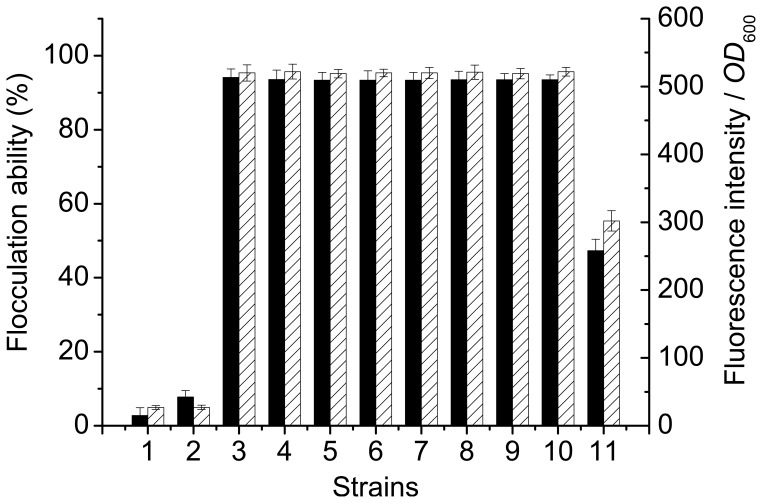
Flocculation ability and fluorescence intensity of YS59, YS58 and recombinant yeast strains. 1: YS58, 2: YSP50, 3: YSF1, 4: YSF1c, 5: YSF1c1, 6: YSF1c2, 7: YSF1c3, 8: YSF1c12, 9: YSF1c13, 10: YSF1c23, 11: YS59. Symbols: flocculation ability (█); fluorescence intensity per *OD*_600_ (▒). Values are means of three independent experiments, and error bars represent standard deviation (n = 3).

The flocculation levels were compared among the donor strain YS59, host strain YS58 and recombinant yeast strains. The expression of *FLO1* and its derived forms in nonflocculent *S.* *cerevisiae* YS58 resulted in almost same degree of flocculation, which was about twofold of that of strain YS59 (Figure 2). As control, strains YS58 and YSP50 displayed no obvious flocculation under same condition. The active flocculins on the surface of yeast cells were determined using Avidin-FITC. In the presence of Ca^2+^, recombinant strains carrying the *FLO1* or its derivatives were able to fix the fluorescent probe Avidin-FITC at similar concentration, which was about 1.7 fold of that bound by strain YS59. In contrast, cells of YS58 and YSP50 were not able to bind Avidin-FITC (Figure 2). The above results indicated that similar levels of flocculin present on the surface of cells of yeast strains YSF1, YSF1c, YSF1c1, YSF1c2, YSF1c3, YSF1c12, YSF1c13 and YSF1c23, which led to almost equal degree of flocculation. This suggests that deletion of the repeats in unit C of *FLO1* might not influence the function of flocculin.

### Physiological Characteristics of Flocculation in Different Yeast Strains

#### Flocculation of yeast strains carrying *FLO1* or its derivatives with partial or complete deletion of repeat unit C was inhibited only by mannose

**Figure 3:**
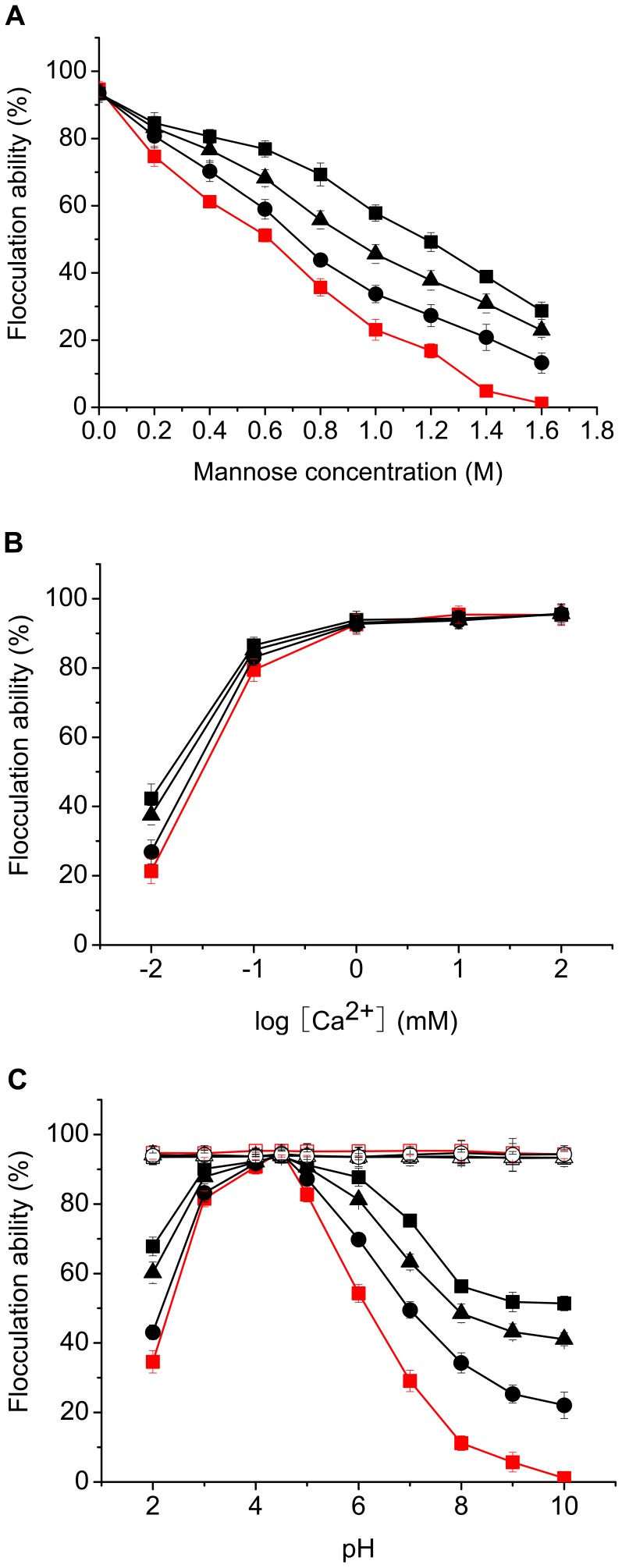
Effect of mannose, Ca^2+^ and pH on flocculation of different yeast strains. Flocculation ability was compared under conditions with different concentrations of mannose (A) and Ca**^2+^** (B) at pH 4.5, and different pH values (C). Symbols: YSF1 (red squares: ▪, □), YSF1c1/YSF1c2/YSF1c3 (black circles: •, ○), YSF1c12/YSF1c13/YSF1c23 (black triangles: ▴, △), YSF1c (black squares: ▪, □), open symbols represent the flocculation recovery of strains treated by different pH conditions. Because almost same levels of flocculation were obtained for the three variants YSF1c1, YSF1c2 and YSF1c3 under same conditions, data for one of the three variants were shown as an example. The same treatment was performed for variants YSF1c12, YSF1c13 and YSF1c23. Values are means of three independent experiments, and error bars represent standard deviation (n = 3).

The flocculation levels of different yeast strains were compared in the presence or absence of sugars. In the presence of 0.5 M of different sugars, the flocculation of all the tested yeast strains was inhibited largely only by mannose (Table 2), and a progressively inhibitory effect on flocculation with increasing mannose concentration was observed for the above strains (Figure 3A). However, the sensitivity of flocculation to mannose showed some quantitative relationship to the number of repeats in unit C: the more repeats, the more sensitive flocculation to mannose. However, the position of deletion in repeat unit C had no effect on sensitivity of flocculation to mannose. In the presence of 1 M mannose, the flocculation of strain YSF1 decreased by 75.6%, while that of strain YSF1c decreased only by 38.1%. At the same condition, flocculation of strains YSF1c1, YSF1c2 and YSF1c3 with one repeat deletion in unit C of *FLO1* decreased by 63.9%, while that of strains YSF1c12, YSF1c13 and YSF1c23 with two repeats deletion in unit C of *FLO1* decreased by 51.1%. Strain YSF1 lost the flocculation ability completely under condition with 1.6 M mannose, while strain YSF1c still displayed 28.7% flocculation ability. In contrast, no obviously inhibitory effect on flocculation was observed for the tested strains in the presence of glucose, galactose, maltose or sucrose, even at high sugar concentrations (data not shown). This indicates that variation of repeat number in unit C might affect the avidity of flocculin to monosaccharide mannose or the conformational change caused by binding of mannose rather than the specificity of carbohydrate recognition of flocculins.

**Table 2:** Inhibition on flocculation of different yeast strains by sugars.

Strains	% Flocculation ability with					
	Nosugar	Mannose	Glucose	Maltose	Sucrose	Galactose
YS59	41.5±1.3	13.2±1.5	38.4±1.6	43.7±1.0	44.4±1.5	39.4±1.2
YSF1	95.1±1.4	55.1±1.8	89.6±2.9	89.9±2.6	90.8±2.2	92.7±1.3
YSF1c1	94.2±1.2	62.3±1.7	90.7±1.4	89.7±1.7	90.4±1.4	91.7±1.3
YSF1c2	94.0±1.4	62.7±1.9	90.3±1.8	90.1±1.2	90.7±1.9	92.3±1.4
YSF1c3	93.9±1.1	61.9±2.3	89.7±1.9	89.6±1.4	90.7±1.3	92.4±1.7
YSF1c12	93.5±1.5	69.1±1.5	90.2±1.5	89.7±1.4	90.8±1.4	92.7±1.9
YSF1c13	93.9±2.1	68.4±1.7	89.9±1.7	90.2±1.7	91.3±1.3	93.1±2.1
YSF1c23	93.3±1.9	68.9±1.3	90.3±1.4	90.2±1.4	91.7±1.7	92.5±2.4
YSF1c	93.9±1.7	78.4±1.1	89.8±1.3	90.7±1.4	91.7±0.8	92.3±0.8

The concentration of each sugar is 0.5 M. Data are means ± standard deviations of three independent experiments.

#### Dependence of flocculation on calcium

The influence of calcium ion on flocculation of yeast strains was investigated. As shown in Figure 3B, all the tested yeast strains displayed the Ca^2+^-dependent flocculation. At Ca^2+^ concentrations below 1 mM, flocculation was induced progressively by increasing concentration of Ca^2+^, which was very obvious at Ca^2+^ concentration below 0.1 mM. When Ca^2+^ concentration was more than 1 mM in the buffer, flocculation of all strains maintained. However, the induction of Ca^2+^ on flocculation of strains with partial or complete deletion of repeat unit C in *FLO1* was greater than on that of YSF1. Strain YSF1c in particular displayed the highest flocculation levels at Ca^2+^ concentration below 1 mM when compared to other strains. This suggests that flocculins with partial or complete deletion of repeat unit C might have higher affinity to Ca^2+^ or have higher conformational stability than Flo1, which need lower concentration of Ca^2+^ than Flo1 to keep in an active conformation.

#### Flocculation endowed by flocculins with partial or complete deletion of repeat unit C displayed a broad tolerance to pH fluctuation

The relationship between pH and flocculation was investigated (Figure 3C). All of yeast strains displayed the maximal flocculation level at pH 4.5. However, significant differences in flocculation level were observed for different strains at pH values fluctuated from 4.5. A pH deviation from 4.5 had significant inhibition on flocculation of strain YSF1, while flocculation of strain YSF1c occurred optimally across a pH range of 3.0–6.0 and was more stable than that of YSF1 between pH 2.0 and 10.0. Flocculation of YSF1 was reduced by 63.3% at pH 2.0 and completely lost at pH 10.0. In contrast, flocculation of YSF1c was reduced by 27.6% at pH 2.0 and 51.4% of flocculation was still preserved at pH 10.0. The inhibitory effects of pH on flocculation of strains YSF1c1 (YSF1c2 or YSF1c3) and YSF1c12 (YSF1c13 or YSF1c23) lay between YSF1 and YSF1c, and were not relevant to the position of deletion. When yeast cells were harvested from flocculation buffers with different pH values and resuspended in 50 mM sodium acetate buffer (pH 4.5), the maximal flocculation levels were recovered for all strains, which indicated that pH fluctuation did not affect the homophilic binding of flocculins with cell wall, but unfavorable pH led to the reversible denaturation of flocculins. Because the only difference existed among these strains is the variation in number of repeat in unit C of flocculins, and the fewer repeats, the stronger adaptability to pH fluctuation, the results suggest that deletion of repeat unit C sequence in *FLO1* might increase the conformational stability of flocculins to pH fluctuation, thus retaining relatively higher flocculating activity than Flo1 across a pH range of 2.0–10.0.

#### Combined effect of pH and Ca^2+^ on flocculation

**Figure 4:**
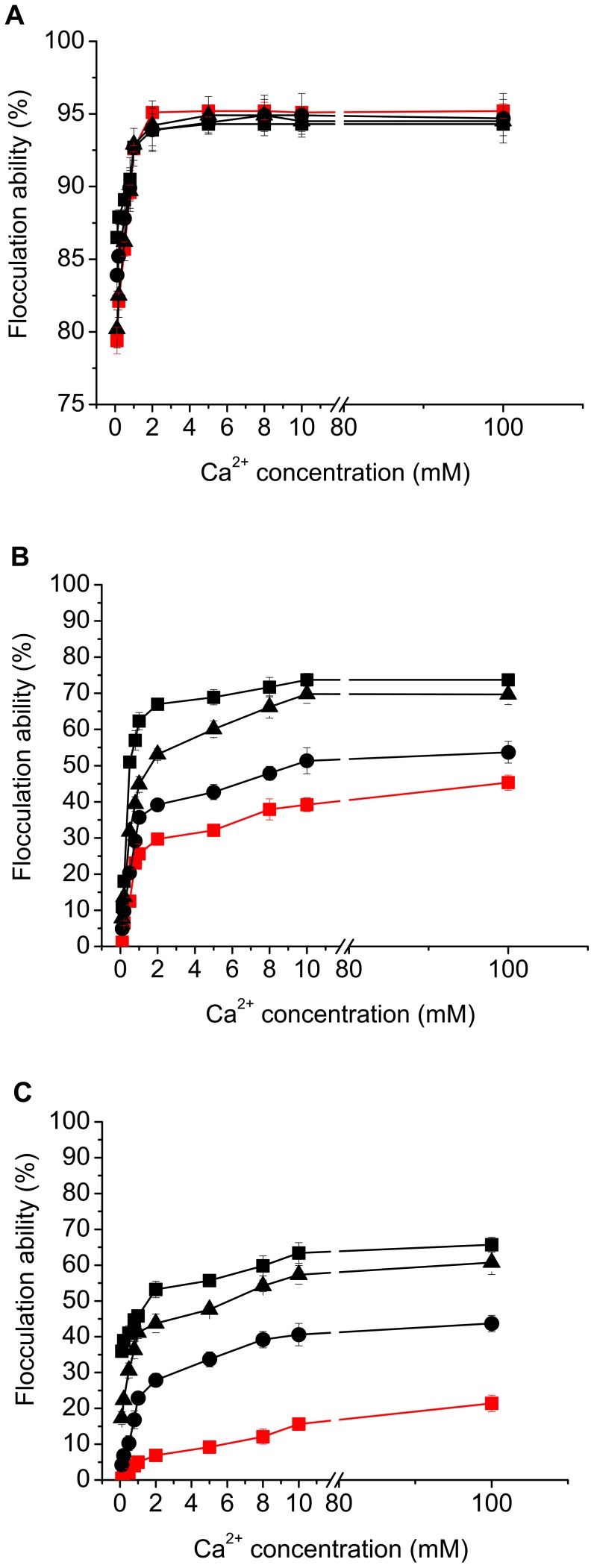
Flocculation abilities of yeast strains under different pH with different Ca^2+^ concentrations. Flocculation was determined as described in Materials and Methods. A. pH 4.5, B. pH 2.0, C. pH 8.0. Symbols: YSF1 (red square: ▪), YSF1c1/YSF1c2/YSF1c3 (black circle: •), YSF1c12/YSF1c13/YSF1c23 (black triangle: ▴), YSF1c (black square: ▪). Because almost same levels of flocculation were obtained for the three variants YSF1c1, YSF1c2 and YSF1c3 under same conditions, data for one of the three variants were shown as an example. The same treatment was performed for variants YSF1c12, YSF1c13 and YSF1c23. Values are means of three independent experiments, and error bars represent standard deviation (n = 3).

The combined effect of pH and calcium ions on flocculation of yeast strains was investigated. As shown in Figure 4, in all tested pH conditions, all strains displayed Ca^2+^-dependent flocculation. In agreement with results shown in Figure 3B, at pH 4.5, all strains exhibited almost similar profiles of flocculation over the whole range of Ca^2+^ concentration, and the maximal flocculation levels were observed at Ca^2+^ concentration of 5 mM (Figure 4A). At pH 2.0, flocculation was provoked with the increase of Ca^2+^ concentration for all strains. However, strain YSF1 displayed much lower flocculation levels than other strains in the Ca^2+^ concentration range investigated (Figure 4B). Moreover, for strains YSF1c1 (YSF1c2 or YSF1c3), YSF1c12 (YSF1c13 or YSF1c23) or YSF1c, 2, 0.8 or 0.4 mM of Ca^2+^ was needed to produce 40% of flocculation at pH 2.0, whereas 10 mM of Ca^2+^ was needed for strain YSF1 to reach such flocculation level. Flocculation levels of strains YSF1 and YSF1c increased by 53.8% and 15.9% respectively at Ca^2+^ concentrations from 1 mM to 10 mM at pH 2.0. When the pH of cell suspension was set at 8.0, although profiles of flocculation change were similar to those at pH 2.0, very low flocculation occurred for strain YSF1 over the whole Ca^2+^ concentration range (Figure 4C). Accordingly, results in Figure 4 confirms that flocculation of YSF1 is more sensitive to pH change than that of strains with partial or complete deletion of repeat unit C in *FLO1*, and basic condition has much severer inhibition on flocculation of YSF1 than acidic condition. Moreover, the inhibitory effect produced by pH change on flocculation can be relieved partially by addition of calcium ions.

Collectively, the variation of repeat number in unit C of *FLO1* influences the sensitivity of flocculation to free mannose and pH fluctuation: the more the repeats, the more the sensitivity. Based on the results of physiological characteristics analyses of flocculation for different strains, we hypothesized that the deletion of repeats in unit C of flocculin Flo1 might increase the conformatial stability of flocculin to pH fluctuation. To validate this, strains YSF1 containing the intact *FLO1* and YSF1c with complete deletion of unit C in *FLO1* were chosen for further investigations.

### Hydrophobicity of Cell Surface at Different pH Values

**Figure 5:**
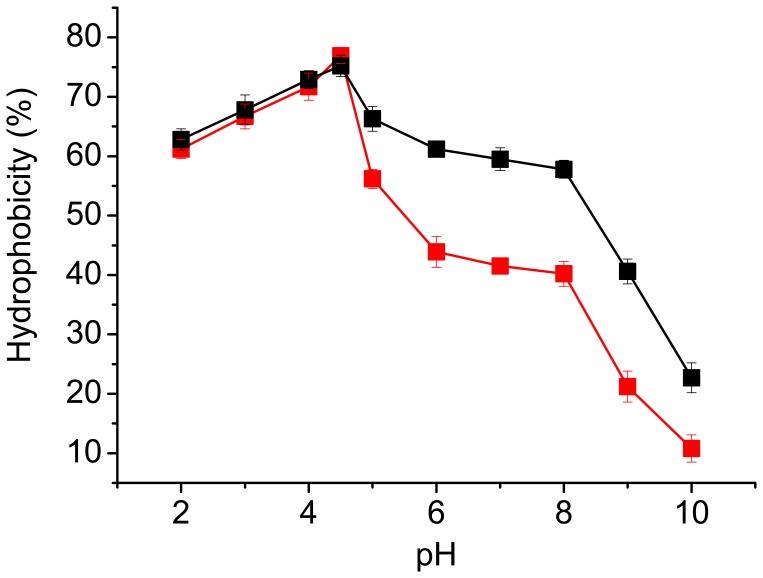
Cell surface hydrophobicity of strains YSF1 and YSF1c at different pH values. The hydrophobicity of cell surface was determined using the method described in Materials and Methods. Symbols: YSF1 (red square: ▪), YSF1c (black square: ▪). Values are means of three independent experiments, and error bars represent standard deviation (n = 3).

Cell surface hydrophobicity of YSF1 and YSF1c under different pH conditions was determined by measuring the distribution ratio of yeast cells in a biphasic system consisting of a buffered solution and an organic solvent. Both strains displayed the maximal hydrophobicity at pH 4.5, and a deviation of pH from 4.5 led to evidently reduction of hydrophobicity (Figure 5), which was very in agreement with the change profiles of flocculation at different pH (Figure 3C). Strain YSF1c exhibited higher hydrophobicity and flocculation levels than strain YSF1 under conditions with pH over 4.5, thus a positive correlation between hydrophobicity and flocculation was observed. Because the production of hydrophobicity is a consequence of the presence of active flocculins on cell surface, the results indicate that larger amount of active flocculins might present on cells of YSF1c than on cells of YSF1 at neutral or alkaline conditions.

### Characterization of the Conformational Stability of Flocculins by Fusion Expression with GFP

**Figure 6:**
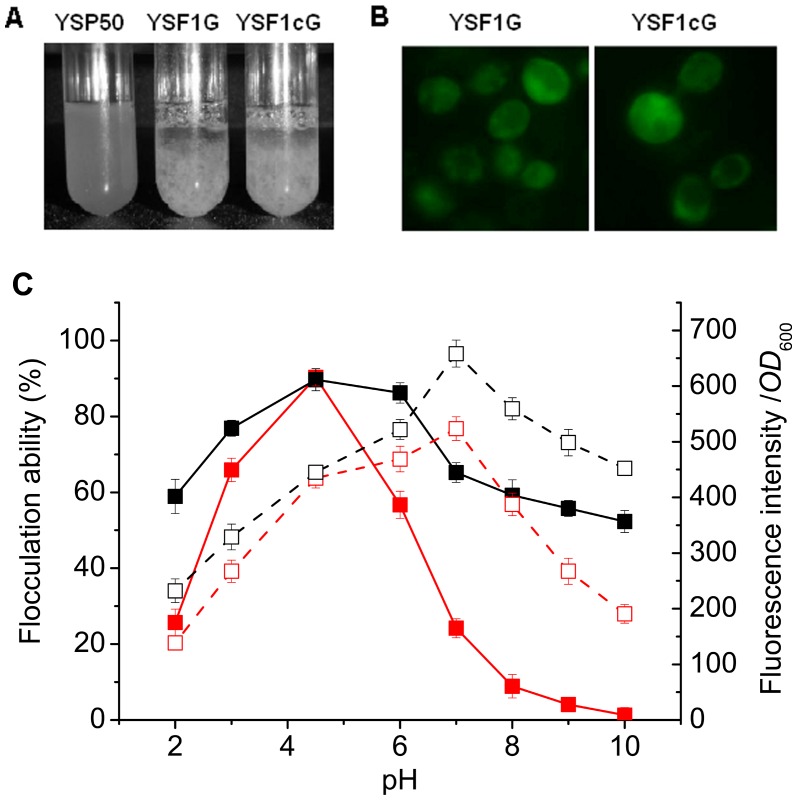
Characterization of conformational stability of flocculins at different pH values by fusion expression with GFP. A. Picture of flocculation of yeast strains YSF1G and YSF1cG taken after growing in YPD at 30°C and 200 rpm for 24 h. B. Presence of GFP at the surface of yeast strains YSF1G and YSF1cG visualized by fluorescence microscopy as described in Materials and Methods. C. Profiles of flocculation ability and GFP fluorescence intensity versus pH for yeast strains YSF1G and YSF1cG. Symbols: flocculation ability of YSF1G (red filled square: ▪), flocculation ability of YSF1cG (black filled square: ▪), fluorescence intensity of YSF1G (red open square: □), fluorescence intensity of YSF1cG (black open square: □). Values are means of three independent experiments, and error bars represent standard deviation (n = 3).

The flocculation of strain YSF1c displayed a broader tolerance to pH change than that of strain YSF1. We suggested that flocculin Flo1c might have higher conformational stability than Flo1 under different pH conditions. To test this speculation, GFP was used as fluorescence based indicator of conformational stability of flocculins at different pH values. The fusion expression cassettes of *GFP5* with *FLO1* (*GFP5-FLO1*) and *FLO1c* (*GFP5-FLO1c*) were constructed using fusion PCR (Figure 1B) and introduced into nonflocculent *S. cerevisiea* YS58 by plasmids pYCF1G and pYCF1cG to generate recombinant strains YSF1G and YSF1cG. Both strains displayed evident flocculation in the exponential and stationary growth phases (Figure 6A), and similar flocculation efficiency was observed for strains YSF1G and YSF1cG at pH 4.5 by subsequently standard flocculation assays (Figure 6C). The GFP fusion proteins were abundantly localized on the cell wall of strains YSF1G and YSF1cG (Figure 6B). Subsequent fluorescence intensity assays demonstrated that strains YSF1G and YSF1cG produced similar amounts of GFP on cell surface (Figure 6C, pH 4.5). This indicates that both GFP and flocculins were functionally expressed in a fusion manner, and a similar level of fusion proteins GFP-Flo1 and GFP-Flo1c was produced in strains YSF1G and YSF1cG.

The fluorescence intensity and flocculation level of strains YSF1G and YSF1cG under different pH conditions were determined. As shown in Figure 6C, both YSF1G and YSF1cG displayed the maximal flocculation ability at pH 4.5, and the profiles of flocculation versus pH similar to that shown in Figure 3C. This indicates that the fusion expression of GFP does not affect the function of flocculins. Strains YSF1G and YSF1cG produced the similar level of fluorescence intensity at pH 4.5, but the maximal fluorescence intensities were observed at pH 7.0 (Figure 6C). The change of fluorescence intensity at different pH resulted from the combined effect of pH on the conformations of GFP and flocculins. Acidic conditions produced inhibitory effect on functionality of both GFP and flocculins leading to the evident reduction of fluorescence intensity, while activation on GFP and inhibition on flocculins occurred at pH values from 4.5 to 7.0 or 10.0. The denaturation of flocculins caused by pH change might influence the conformation of the fused GFP negatively, which cut down the activation of GFP by pH increase. With the increase of pH at alkaline conditions, the inhibitory effect on GFP resulted from denaturation of flocculins increased and overtook the activation, thus declines of fluorescence intensity gradually were observed. However, strain YSF1cG exhibited much higher fluorescence intensity than strain YSF1G over the whole pH range investigated other than 4.5. This indicates that fusion protein GFP-Flo1c has higher stability than GFP-Flo1 to pH change, which reflects that flocculin Flo1c has more stable conformation than Flo1 to pH fluctuation in environment.

## Discussion

Four families of repetitive sequences (repeat units A, B, C and D) are identified in the central domain of *FLO1* according to the similarities of encoding amino acid sequences, and the fact that number variation of repeats in unit A of *FLO1* gene creates quantitative alternations in phenotypes (flocculation, adhesion, or biofilm formation) or specificity for sugar recognization was discovered [14], [17]. Meanwhile, complete deletion of unit B or unit D in *FLO1* increased the tolerance of flocculation of yeast cells to pH or mannose variation [22]. However, no report about the influence of repeat unit C on flocculation has been released, and that remains an interesting question to be explored.

In this study, the intact *FLO1* gene and its derived forms with partial or complete deletion of repeat unit C were expressed in a nonflocculent *S. cerevisiae* strain YS58, and a similar level of Flo1-type flocculation was produced. The deletion of repeat unit C did not influence the sugar-binding strength and specificity of flocculin, which is different from the previous reports about the relationship of flocculation strength with size variation of *FLO1* [14], [17]. This indicates that repeat unit C is different from repeat unit A for regulation of function of flocculins. However, free mannose produced different degrees of inhibition on the flocculation of the yeast strains, and more mannose was required for complete inhibition of flocculation endowed by flocculins with deletion of repeats in unit C than by Flo1, which is very consistent with our previous study about deletion of repeat unit D in *FLO1* [22]. The similar level of flocculins on cell surface, as well as the similar flocculation ability among these strains indicates that there is no evident difference for Flo1 and its derivatives in affinity to oligosaccharide mannose on yeast cell wall. It has been found that Flo5A has much higher affinity to α1,2-linked mannosides than to monosaccharide mannose in vitro, and different conformation alternations were induced by binding with mannose or α1,2-linked mannosides [23]. Accordingly, we suggest that deletion of repeat unit C in the central domain of flocculin Flo1 does not influence the avidity of protein to α1,2-linked mannosides at cell surface, but might increase the conformation stability of flocculin, which makes it possible for the mannose-bound flocculins to bind and react with α1,2-linked mannosides at the surface of adjacent cells leading to higher levels of flocculation than Flo1 in the presence of mannose.

Flocculation is a calcium-dependent physiological process. The role of calcium in flocculation has been clarified as direct involvement in carbohydrate binding and stabilizing the conformation of flocculins by interaction with the glycosylated central domains [23], [24], [25]. Deletion of repeat unit C in the central domain of flocculin Flo1 did not influence the dependence of flocculation on calcium, but increased the sensitivity to calcium. Fewer calcium ions were required for strains with deletion of repeats in unit C of *FLO1* than strain YSF1 to obtain the same levels of flocculation. This further suggests that deletion of repeat unit C increases the conformation stability of flocculin.

The impact of pH fluctuation on flocculation might result from changes of electrostatic charge on cell surface, proteins conformation or *FLO* gene expression [26]. All the tested strains displayed the maximal flocculation ability at pH 4.5, and pH fluctuation from 4.5 produced significant inhibition on flocculation. However, flocculation conferred by flocculins with deletion of repeats in unit C displayed a broader tolerance to pH fluctuation than by Flo1, and the fewer the repeats in unit C, the stronger adaptability of flocculins to pH fluctuation. The tolerance of flocculation to pH fluctuation was also improved by complete deletion of unit B or D in *FLO1* [22], however, which was less obvious than deletion of unit C in *FLO1*. Under condition with pH value of 2.0, yeast strains with complete deletion of unit B, D or C in *FLO1* displayed 42.3%, 56.2% or 67.7% of flocculation ability respectively, while at pH of 7.0, flocculation ability preserved at 57.8%, 61.2% or 76.6% for the above strains. Because a similar level of flocculins was detected on cell surface of different yeast strains (Figure 2), and no obvious changes in electrostatic charge on cell surfaces were observed at different pH conditions (data not shown), the reduction of flocculation ability caused by pH fluctuation from 4.5 maybe due to conformational change of flocculins. Confirmation of the reversible denaturation of flocculins under unfavourable pH conditions (Figure 3B) and the partial relief of the inhibitory effect on flocculation caused by pH fluctuation by addition of calcium ions (Figure 4) further led to the conception that pH fluctuation causes conformational changes for flocculins in this study. The hydrophobicity on cell surface is a consequence of the presence of active flocculins on the yeast cell wall, and a positive correlation between the cell hydrophobicity and active flocculins was found [27], [28], [29]. The evident reduction of hydrophobicity on cell surface under conditions with pH fluctuation from 4.5 confirms the conformation alteration of flocculins. Yeast strain YSF1c displayed higher hydrophobicity than strain YSF1 at neutral and alkaline conditions, which suggested that slighter conformational alteration occurred for flocculin Flo1c as compared to Flo1 under unfavourable pH conditions. This indicates that flocculin Flo1c might have higher conformational stability than Flo1 to pH fluctuation.

Green fluorescent protein (GFP) has been extensively used as gene-based probes in molecular and cell biology. The fluorescence of GFP was lower at acidic conditions than at physiological and alkaline conditions [30], and the photophysical characteristics of GFP remain relatively stable at alkaline conditions [31]. In this work, GFP encoded by *GFP5* was used as the indicator of influence of pH on flocculin conformation by fusion expression. The similar levels of fluorescence and flocculation displayed between strains YSF1G and YSF1cG at pH 4.5, the optimal pH for flocculation of yeast cells, indicated that equal amounts of fusion proteins GFP-Flo1 and GFP-Flo1c were produced. The fluorescence dependence on pH might result from the combined effect of pH on the states of GFP chromophore, the GFP conformation and the flocculin conformation. Under conditions with pH values below 4.5, both fluorescence and flocculation of strains YSF1G and YSF1cG decreased largely for the conversion of GFP chromophore from anionic state to neutral state [31], the conformational alterations of GFP and flocculins. A rise in pH from 4.5 triggered the gradually conversion of GFP chromophore from neutral state to anionic state, the formation of active conformation for GFP and denaturation of flocculins. The dynamic changes in significance of the above three effects at different pH values led to the increase or decrease of fluorescence. Because the effects of pH on the states of GFP chromophore and GFP conformation are same between fusion proteins GFP-Flo1 and GFP-Flo1c, the difference of fluorescence intensity between YSF1G and YSF1cG at pH values fluctuating from 4.5 should be attributed mainly to conformational alteration of flocculins. The higher levels of fluorescence displayed by strain YSF1cG than strain YSF1G across the pH range of 2.0–10.0 except 4.5, as well as the similar level of fluorescence displayed by YSF1G and YSF1cG at pH 4.5, proved that fusion protein GFP-Flo1 is more sensitive to pH than GFP-Flo1c. The only difference between GFP-Flo1 and GFP-Flo1c is the existence or absence of repeat unit C in the central domain of flocculins. Accordingly, deletion of repeat unit C in the central domain increases the conformational stability of flocculin to pH fluctuation.

In conclusion, variation of the number of tandem repetitive sequences in the central domain of flocculins can alter the functional properties of proteins. Repeat unit C is the second set of repetitive sequence in size in the central domain of flocculin Flo1 which comprised of three repeats of 51 amino acid residuals and an internal space of 14 amino acid residuals. We hypothesized initially that deletion of repeat unit C in Flo1 truncates the flocculin, which might lead to the reduction of flocculation ability as reported for repeat unit A [17] or alteration of other properties of flocculation [14], [22]. Consistently with the results of deletion of unit B or unit D other than unit A, no obvious differences in flocculation ability and specificity of carbohydrate recognition were observed for yeast strains carrying the intact *FLO1* or the derived forms with partial or complete deletion of repeat unit C respectively, which indicates the truncated flocculins can stride across the cell wall and cluster the N-terminal domain on yeast cell surfaces as the intact Flo1 and thereby improving intercellular binding. Moreover, deletion of repeats in unit C of *FLO1* increased the tolerance of flocculation to free mannose and pH fluctuation as well, and the fewer the repeats in unit C, the stronger adaptability of flocculation to environmental changes, which was not relevant to the site of deletion. Combined with the further analyses of hydrophobicity on the surface of yeast cells and stability of the GFP conformation fused to Flo1 and Flo1c, we propose that more stable active conformation can be obtained for flocculin by deletion the repeat unit C in the central domain of *FLO1*. As the flocculation characteristics of yeast has great industrial significance, the broad tolerance of flocculation to environmental stress, especially pH fluctuation, supports its extensive application in industrial fermentation process and environmental remediation.

## Materials and Methods

### Strains, Plasmids and Culture Media

The typical Flo1-type flocculent strain of *S*. *cerevisiae* YS59 (*MATα FLO1 ura3-52 leu2-3,112 his4-519 trp1-789*) was used as the donor of *FLO1* gene and the positive control during physiological tests. The non-flocculent strain of *S. cerevisiae* YS58 (*MATα flo1 ura3-52 leu2-3,112 his4-519 trp1-789*) was used as the host strain for expression of *FLO1* gene and its derived forms [32]. *Escherichia coli* DH5α (*SupE44, hsdR17, recA1, ndA1, gyrA96, thi-1 relA1, ΔLacU169*(*φ80LacZΔ M15*)) was used for plasmid construction and maintenance. The *E. coli*-yeast centromeric shuttle plasmid YCp50 (*amp*, *ARS1*, *CEN4*, *URA3*) was used for recombinant plasmid construction. Plasmid pGEM-T-GFP carries the modified green fluorescent protein gene *GFP5*. Yeast cells were cultured at 30°C in yeast peptone dextrose medium (YPD), or minimal synthetic defined medium (SD) supplemented with the necessary amino acids and bases [33]. *E. coli* cells were cultivated at 37°C in Luria-Bertani (LB) medium [34]. For plasmid selection, ampicillin was added to the LB medium to a final concentration of 50 μg/ml. Solid media were prepared by adding 1% (w/v) agar to the liquid media.

### DNA Manipulation

Standard DNA manipulation in *E. coli* was performed as described by Sambrook and Russell [34]. The isolation of yeast chromosomal DNA and transformation of yeast cells were carried out using the methods described by Adams *et al*. [33]. Plasmid was isolated from yeast cells using the yeast plasmid kit (OMEGA Bio-Tek, USA) according to the described protocol. Polymerase chain reaction (PCR) was conducted using high fidelity DNA polymerase KOD plus (TOYOBO, Japan) according to the described protocol.

### Primers and Plasmid Construction

Primers used in this study were described in Table 1 and Figure 1. The intact *FLO1* (EF670005.1) was amplified from the genomic DNA of *S. cerevisiae* YS59 by PCR with a primer set of P1 and P4. The 6082 bp PCR product contains the intact ORF, the upstream regulation sequence, and the downstream sequence of the *FLO1* gene. To obtain the derived form of *FLO1* with complete deletion of repeat unit C, primers P2 and P3, which correspond to the flanking sequences of repeat unit C, were designed (Figure 1A). There was a 29 bp complementary sequence between primers P2 and P3. Using the intact *FLO1* as template, 4345 bp DNA fragment containing the upstream sequence of repeat unit C in *FLO1* was amplified by PCR with primer pairs P1 and P2, and 1236 bp DNA fragment containing the downstream sequence of repeat unit C in *FLO1* was amplified with primer pairs P3 and P4. The derived form of *FLO1* with complete deletion of repeat unit C was obtained by fusion PCR with primers P1 and P4 using above 4345 bp and 1236 bp DNA fragments as co-templates and designated as *FLO1c*. Derivatives of *FLO1* with partial deletion of unit C were obtained by fusion PCR using primers listed in Table 1, which were designated as *FLO1c1* with C1 deletion, *FLO1c2* with C2 deletion, *FLO1c3* with C3 deletion, *FLO1c12* with C1 and C2 deletion, *FLO1c13* with C1 and C3 deletion, and *FLO1c23* with C2 and C3 deletion respectively (Figure 1A). Sequences of *FLO1* and its derivatives were analyzed and verified using DNAMAN sequence analysis software (Lynnon Biosoft, Canada). The *Sal*I-*Hin*dIII digested *FLO1* and its derivatives were inserted into *Sal*I and *Hin*dIII sites in plasmid YCp50 to generate recombinant expression plasmids pYCF1, pYCF1c, pYCF1c1, pYCF1c2, pYCF1c3, pYCF1c12, pYCF1c13, and pYCF1c23 respectively.

To construct plasmids for expression of green fluorescence protein (GFP) tagged flocculins, primers P5, P6, P7 and P8 were designed (Table 1, Figure 1B). There were 40 bp of complementary sequence between P5 and P6, and 29 bp between P7 and P8 respectively. The open reading frame (714 bp) without termination codon of *GFP5* was obtained from plasmid pGEM-T-GFP by PCR using primers P6 and P7. The promoter and signal sequence of *FLO1* was amplified with primers P1 and P5 and designated as *P1P5*. A fusion DNA fragment containing the promoter, signal sequence of *FLO1* and *GFP5* was obtained by fusion PCR with primers P1 and P7 using *P1P5* and *GFP5* as co-templates and designated as *P1P7*. The terminator sequence and coding sequences except signal sequence of *FLO1* or *FLO1c* were obtained from plasmids pYCF1 or pYCF1c by PCR with primers P8 and P4, which were designated as *FLO1-P8P4* and *FLO1c-P8P4* respectively. The fusion expression cassettes of *GFP5* with *FLO1* or *FLO1c* were obtained by fusion PCR with primers P1 and P4 using *P1P7* and *FLO1-P8P4* or *FLO1c-P8P4* as the co-templates and designated as *GFP-FLO1* and *GFP-FLO1c* respectively. The *Sal*I-*Hin*dIII digested *GFP-FLO1* and *GFP-FLO1c* were inserted into *Sal*I and *Hin*dIII sites in plasmid YCp50 to generate recombinant expression plasmids pYCF1G and pYCF1cG respectively.

### Yeast Transformation and Genetic Stability Analysis of Recombinants

The nonflocculent *S. cerevisiae* YS58 was transformed with empty vector YCp50 and different recombinant plasmids using the lithium acetate method [33]. The transformed yeast cells were screened using uracil-auxotrophic complementation on SD medium containing leucine, histidine and tryptophan. For analysis of genetic stability of recombinant strains, yeast cells were grown in YPD medium for 10 generation. Each generation of the strain was cultivated at 30°C for 24 h. Yeast cells from the tenth generation were diluted and spread on YPD plates. After cultivation for 48 h at 30°C, 100 of single colonies were chosen and analyzed for their auxotroph.

### Flocculation Assay

Flocculation assay was performed as described previously [12], with some modifications. Yeast cells were grown in 2 ml of YPD at 30°C with shaking (200 rpm) for 18 h. 1.5 ml of the culture was then inoculated to 150 ml of YPD in 500 ml shake flasks and cultured for 24 h under the same condition. Yeast cells were harvested by centrifugation at 3,000×*g* for 5 min and divided into various equal parts. After washed twice with 50 mM EDTA (pH 8.0) and sterile water respectively, yeast cells were resuspended in 5 ml of appropriate flocculation buffers with or without 6.8 mM CaCl_2_ to a final concentration equivalent to an *OD*_600_ of 1.8. After 5 min of vigorous agitation and 5 min of stationary cultivation at 30°C, 3 ml of the upper phase was withdrawn and measured at 600 nm. Flocculation ability was determined by the equation F = (1–B/A)×100%, where *F* represents the flocculation ability, *A* is the *OD*_600_ without Ca^2+^ and *B* is the *OD*_600_ with Ca^2+^. For general assay of flocculation, 50 mM sodium acetate buffer (pH 4.5) was used. To assess the influence of pH on flocculation, different buffers were used: 50 mM sodium acetate buffer for pH 2.0 to 7.0, and 10 mM tris base buffer for pH 8.0–10.0. All the tests were repeated three times under same conditions.

### Determination of Active Flocculins on Yeast Cell Surface

The fluorescent probe Avidin-fluorescein isothiocyanate (Avidin-FITC) was used to detect the active flocculins on yeast cell surface as described previously [29], [35], with minor modification. Yeast cells were cultured, harvested and washed as the flocculation assay, and resuspended in 50 mM sodium acetate buffer (pH 4.5) to a final concentration equivalent to an *OD*_600_ of 0.5. Avidin-FITC (Sigma-Aldrich, USA) and CaCl_2_ were successively added to the cell suspension at a final concentration of 10 µg/ml and 6.8 mM respectively. The mixture was vortexed vigorously for 10 s and incubated at 25°C for 30 min in dark. The fluorescence intensity of mixture was detected using a Bio-Tek Synergy HT Multi-Mode Microplate Reader (Winooski, USA) with an excitation wavelength of 494 nm, and an emission wavelength of 520 nm run by BioTek Gen5 Data Analysis Software. The mixture was centrifuged at 850 × *g* for 3 min, and the supernatant was withdrawn and measured for fluorescence intensity. As a control, mixtures without Avidin-FITC or CaCl_2_ were also incubated and measured as above. The difference in fluorescence intensity between mixture and supernatant was used to indicate the probe concentration bound by yeast cells for the presence of active flocculins on cell surface. All the tests were repeated three times under same conditions.

### Fluorescence Microscopy and Fluorescence Measurement for GFP

Yeast cells were cultivated and treated as the flocculation assay. Yeast cells were resuspended in sterile water to a final concentration equivalent to an *OD*_600_ of 0.5. The GFP-tagged flocculins were observed using a fluorescence microscopy of Axio Imager A1 (Zeiss, Germany). Images were acquired with AxioCam MRM and processed using AxioVision Rel.

For determination of the GFP fluorescence intensity, yeast cells were resuspended in 3 ml of 50 mM sodium acetate buffer (pH 2.0 and 4.5) or 10 mM tris base buffer (pH 7.0, 8.0 and 10.0) to a final concentration equivalent to an *OD*_600_ of 1.0. After 30 s of vigorous agitation and 5 min of stationary cultivation at 30°C, the fluorescence intensity on yeast cell surface was detected using the BioTek Synergy HT Multi-Mode Microplate Reader with an excitation wavelength of 488 nm and an emission wavelength of 533 nm. All the tests were repeated three times under same conditions.

### Measurement of Hydrophobicity of Yeast Cell Surface

The hydrophobicity of yeast cell surface was determined by measuring the distribution ratio of yeast cells in a biphasic system consisting of a buffered solution and an organic solvent as described previously [23], [28], with some modifications. Yeast cells grown in YPD medium at 30°C with shaking (200 rpm) for 24 h were harvested, washed with 50 mM EDTA (pH 8.0) to deflocculate and resuspended in 50 mM EDTA (pH 8.0) to achieve an *OD*_600_ about 1.0, which was recorded as *I*. Yeast cells from a 3 ml aliquot of this suspension were harvested and resuspended in 3 ml of appropriate buffer. The yeast suspension was overlaid by 1 ml of a hydrophobic hydrocarbon, octane, and vortexed at maximum speed for 1 min and left to stand for 10 min. Aqueous phase was withdrawn carefully and the absorbance at 600 nm was measured and recorded as *F*. The hydrophobicity was calculated as the average modified hydrophobic index (MHI) using the equation: MHI = (1– F/I) × 100%. To analyze the hydrophobicity of yeast cell surface under different pH conditions, buffers with different pH were used: 50 mM sodium acetate buffers for pH 2.0 to 7.0, and 10 mM tris base buffers for pH 8.0–10.0. All the tests were repeated three times under same conditions.
